# Relaxation and Simplex mathematical algorithms applied to the study of steady-state electrochemical responses of immobilized enzyme biosensors: Comparison with experiments

**DOI:** 10.1016/j.jelechem.2008.01.006

**Published:** 2008-05-01

**Authors:** V. Flexer, K.F.E. Pratt, F. Garay, P.N. Bartlett, E.J. Calvo

**Affiliations:** a INQUIMAE, Departamento de Quimica Inorganica, Analitica y Quimica Fisica, Facultad de Ciencias Exactas y Naturales, Universidad de Buenos Aires, Buenos Aires, Argentina; b City Technology Ltd., Walton Road, Portsmouth, Hants PO6 1SZ, UK; c INFIQC, Departamento de Fisicoquímica, Facultad de Ciencias Químicas, UNC, Pab. Argentina 2° piso, Ciudad Universitaria, Córdoba 5000, Argentina; d School of Chemistry, University of Southampton, Southampton, Hants SO17 1BJ, UK

**Keywords:** Electrostatic self-assembly, Enzyme electrodes, Relaxation method, Simplex algorithm

## Abstract

A description of the implementation of the relaxation method with automatic mesh point allocation for immobilized enzyme electrodes is presented. The advantages of this method for the solution of coupled reaction–diffusion problems are discussed. The relaxation numerical simulation technique is combined with the Simplex fitting algorithm to extract kinetic parameters from experimental data. The results of the simulations are compared to experimental data from self-assembled multilayered electrodes comprised of glucose oxidase (GOx) and an Os modified redox mediator and found to be in excellent agreement.

## 1 Introduction

For amperometric enzyme electrodes with the enzyme entrapped in a film, the interplay between diffusion and kinetics results in highly non-linear differential equations for which there are no closed form analytical solutions. Approximate analytical solutions for selected limiting cases have been derived [1,2]. However, it is very useful to complement these with fast and reliable numerical simulations which can treat the intermediate cases.

The main advantages of numerical simulation are that it can be used to calculate the amperometric response over the whole range of experimental parameters and that it gives calculated concentration profiles for the different species within the film. In these respects it complements the approximate analytical treatments available in the literature that are only valid under certain limiting conditions, *i.e.* for limited ranges of the experimental variables. Numerical techniques are particularly valuable for exploring the behaviour of the system in the regions between the analytical limiting cases where, typically, two parameters for the system are comparable and a limiting behaviour is not well established.

Ideally the most powerful approach is to combine the use of approximate analytical solutions that provide physical insight into the nature of the rate limiting processes and the physical behaviour of the system with numerical approaches that can be used to fit the full range of experimental data and to extract the best estimates of the controlling kinetic parameters. For amperometric enzyme electrodes this offers the possibility of rational electrode design where the model is used to predict the amperometric response of the electrode [3]. It also offers the possibility to investigate detailed questions about the operation of the electrode such as the true activity of the immobilised enzyme or the direct determination of the rate of mediated electron transfer to the enzyme within the film – issues which are highly relevant to the optimal design of biosensors and efficient biofuel cell electrodes.

Bartlett and Pratt [1] used numerical simulations to validate their approximate analytical solutions and to investigate the boundary regions between the different limiting cases in a clear example of how numerical simulations and approximate analytical techniques can complement each other in the modelling of a particular physico-chemical system.

Fitting experimental data to theory, both approximate analytical treatments and numerical simulations, is essential both to test the validity of the model and to extract the relevant kinetic constants such as the various rate, diffusion and partition coefficients. This is most effectively achieved using non-linear least squares optimization to fit the experimental data to the theory. To achieve this in a reasonable time using a numerical model requires a computationally efficient numerical simulation algorithm since the simulations will have to be repeated many times during the iterative fitting of the kinetic parameters.

Several numerical techniques have been used to solve both the transient and the steady-state situations with various boundary conditions. For a survey of the early literature see [3]. More recently, the problem of amperometric enzyme electrodes, has been analysed by digital simulation in several papers [1,4–15]. As far as we are aware, Bartlett and Pratt [1] are the only ones to have used the relaxation method [16] to treat an electrochemical problem. The advantage of the relaxation method is that it gives a rapid and stable solution for the steady-state behaviour of strongly coupled non-linear differential equations. In their earlier work Bartlett and Pratt did not describe the implementation of the technique in detail. However, they found excellent agreement between their simulations and the calculations from their approximate analytical treatment although they did not compare their simulations with experimental.

In this paper, we build on and significantly extend the work of Bartlett and Pratt. We describe in detail the relaxation method and its implementation to solve the coupled non-linear kinetic-diffusion problem for an amperometric enzyme electrode. We show how the relaxation method can be combined with the Simplex method [16], in order to fit experimental data and extract the relevant kinetic parameters. We show that the simulations are in good agreement with experimental data from electrostatically self-assembled multilayers of Glucose Oxidase (GOx) and an Os modified polyelectrolyte (PAH-Os), and we use our combined relaxation simulation and Simplex optimisation to determine kinetic data for the system.

This new treatment should be applicable to enzymes immobilized in modified polymers, in redox polymer films [17], in redox hydrogels [18], by antigen–antibody interaction (for example avidin–biotin) [19–21], and electrostatically self-assembled systems in general [22,23]. The treatment is equally valid for both thin monolayers as well as thick multilayered films and offers an efficient approach to the treatment of experimental data. The analysis presented here, together with appropriate approximate analytical formulae can be used in the design and optimization of enzyme electrodes for analytical and biofuel cell applications when the final objective is to obtain a detailed understanding of the operation of the electrode and to be able to predict the response.

## 2 The model

**Fig. 1:**
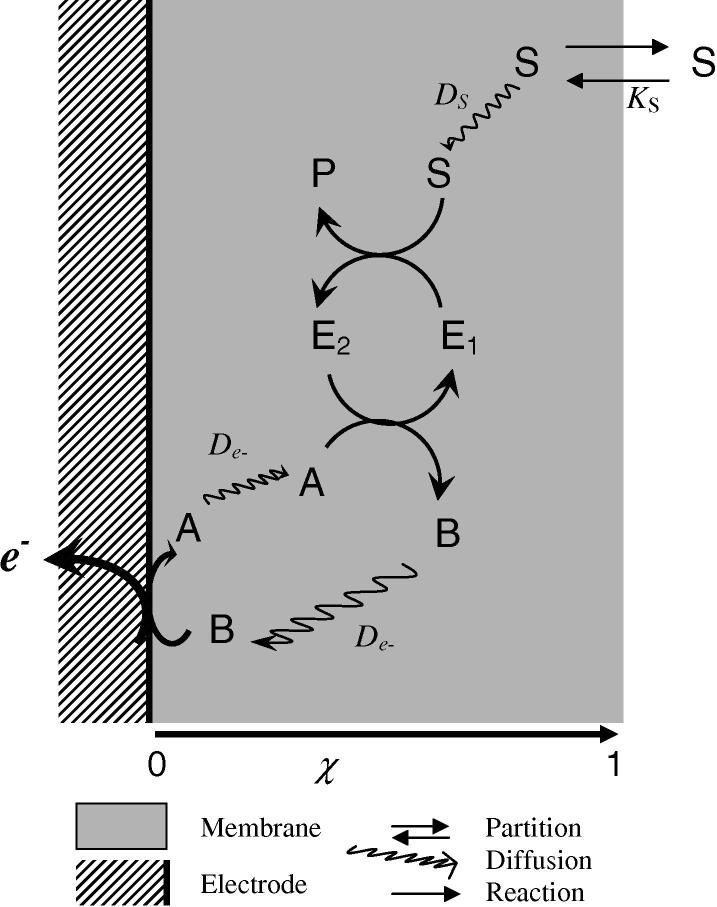
Schematic of a typical enzyme-membrane/electrode showing the processes considered in the model. Diffusion of mediator (A) and substrate (S) occurs within the film with diffusion coefficients $D_{{\text{e}}^{-}}$ (electron hopping) and *D*_s_ respectively. Partition of substrate between the film and the bulk solution is described by the partition coefficient *K*_s_. The homogeneous enzyme kinetics occurs throughout the film from *χ* = 0 to *χ* = 1. The reduced mediator is reoxidized to produce A at the electrode surface. E_1_ and E_2_ are the oxidized and reduced enzyme respectively.

A general kinetic model for an enzyme-membrane electrode has been described previously [1] and is briefly reviewed in Fig. 1 . For fuller details the reader is referred to the original paper. The model describes the general situation of a redox mediated immobilized enzyme electrode. In principle the redox mediator could be co-immobilized in the film or it could be present in the bulk solution and undergo partition into the film. In the latter case, the mediator could be present in the bulk solution either in its oxidized or reduced form. In the present paper we only consider the situation where the mediator is immobilized within the film. This is a very common situation exemplified by, for example, the popular and successful approach of using a redox hydrogel pioneered by Heller and his colleagues [24]. The extension of our treatment to either of the other two situations can be achieved by an analogous numerical treatment taking account of the appropriate boundary conditions [3].

The reactions occurring in the film can be written

(1) $$\text{A}+{\text{E}}_{2}\overset{k}{\to }\text{B}+{\text{E}}_{1}$$

(2) $${\text{E}}_{1}+\text{S}\overset{K_{\text{MS}}}{↔}\text{ES}\overset{k_{\text{cat}}}{\to }{\text{E}}_{2}+\text{P}$$

and at the electrode

(3) $$\text{B}\to \text{A}+{\text{e}}^{-}$$

where A and B are the oxidized and reduced forms of mediator, E_1_ and E_2_ are the oxidized and reduced forms of enzyme, S and P are the substrate and product of the enzymatic reaction and ES is the complex formed between enzyme and substrate (see Appendix 1 for a full list of the symbols used in this work).

The substrate undergoes partition between the bulk solution and the film (partition coefficient *K*_S_) and then diffuses within the film with a diffusion coefficient *D*_S_. The mediator is assumed to be confined within the film. Either it can physically diffuse within the film described by a diffusion coefficient *D*_A_ or, if the mediator is covalently bound within the film, charge propagation occurs by electron hopping self-exchange between the reduced and oxidized dorms of the mediators described by a diffusion coefficient *D*_e_. According to the Dahms–Ruff formalism [25,26] these two situations are equivalent with *D*_A_ = *D*_e_. Michaelis Menten kinetics are assumed for the enzyme–substrate reaction. The oxidized mediator regenerates the enzyme with a mediator–enzyme reoxidation constant *k*, according to the conventional “ping-pong” mechanism [27].

The second-order differential equations describing the system in the steady-state are

(4) $$D_{\text{A}}\frac{d^{2}[\text{A}]}{dx^{2}}=\frac{{\mathrm{kk}}_{\text{cat}}[\text{A}][\text{S}]{\left[\text{E}\right]}_{\text{TOT}}}{k[\text{A}](K_{\text{MS}}+[\text{S}])+k_{\text{cat}}[\text{S}]}$$

(5) $$D_{\text{S}}\frac{d^{2}[\text{S}]}{dx^{2}}=\frac{{\mathrm{kk}}_{\text{cat}}[\text{A}][\text{S}]{\left[\text{E}\right]}_{\text{TOT}}}{k[\text{A}](K_{\text{MS}}+[\text{S}])+k_{\text{cat}}[\text{S}]}$$

The symbols in brackets refer to the concentrations of the corresponding species. These concentrations vary the position within film. Eqs. (4) and (5) are non-linear second order differential equations and have no closed form analytical solution. In this paper we solve these equations using the relaxation method.

First we recast the problem in terms of the following dimensionless variables [1,3]

(6) $$\kappa =L\sqrt{\left(\frac{k{\left[\text{E}\right]}_{\text{TOT}}}{D_{\text{A}}}\right)}$$

(7) $$\eta =\frac{D_{\text{S}}{\mathrm{kK}}_{\text{MS}}}{D_{\text{A}}k_{\text{cat}}}$$

(8) $$\gamma =\frac{{\mathrm{kK}}_{\text{A}}{\left[\text{A}\right]}_{\text{TOT}}K_{\text{MS}}}{k_{\text{cat}}K_{\text{S}}{\left[\text{S}\right]}_{\infty }}$$

(9) $$\mu =\frac{K_{\text{S}}{\left[\text{S}\right]}_{\infty }}{K_{\text{MS}}}$$

(10) $$a=\frac{\left[\text{A}\right]}{{\left[\text{A}\right]}_{\text{TOT}}}$$

(11) $$s=\frac{\left[\text{S}\right]}{K_{\text{S}}{\left[\text{S}\right]}_{\infty }}$$

(12) $$\chi =\frac{x}{L}$$

## 3 Numerical simulations

To analyse the steady-state enzyme electrode response we need only simulate the steady-state solution to Eqs. (4) and (5). The high rates of reaction, and thus steep concentration gradients which can occur within the immobilized layer at the electrode surface, make simulations using an explicit finite difference method impractical. Implicit or semi-implicit methods suffer the drawback that the complex kinetic scheme cannot be solved directly. Consequently we chose to use a steady-state simulation method. The obvious choice, the shooting method [16], is inappropriate in the present case because of the combination of boundary conditions at the two membrane interfaces - the electrode surface and the outer surface of the film. On the other hand, the relaxation method [16] can simultaneously solve any number of coupled first order differential equations, provided that sufficient boundary conditions are known. Eqs. (4) and (5) contain second order diffusional terms but this is not a problem since second (or higher) order differential equations can be expressed as a combination of first order terms.

In the relaxation method a set of ordinary differential equations is replaced by a set of approximate finite difference equations on a grid of points which spans the domain of interest, in this case, the enzyme layer. The principle behind the relaxation method is to start with an initial guess for the concentration profiles within the enzyme layer. The method then computes the resulting errors at each grid point and uses these computed errors to make an improved guess. Hence the name since it could be said that the algorithm *relaxes* to the correct solution. However it is important to note that these intermediate solutions, generated en route to the final steady-state solution have no physical significance and that the final solution is independent of the initial guess.

To illustrate the method consider the trivial case of a single equation **W** in terms of a variable **y**

(13) W(y)=f(y)=0

We starting with an initial guess for the value of **y** and this is adjusted iteratively by a small value **Δy**. For the correct solution

(14) W(y+Δy)=0

Alternatively this can be written

(15) W(y+Δy)=W(y)+VΔy=0

where

(16) $$V=\frac{\partial W(y)}{\partial y}$$

Rearranging Eq. (16) therefore gives the value of **Δy** in terms of the previous estimate for **y**:

(17) $$\Delta y=\frac{W(y)}{V}$$

When dealing with a large number of coupled equations and variables **V** is a matrix containing the differentials of all equations **W** with respect to each variable **y**. Solution of the equations therefore requires inversion of this matrix. Further details are given by Press et al. [16].

**Fig. 2:**
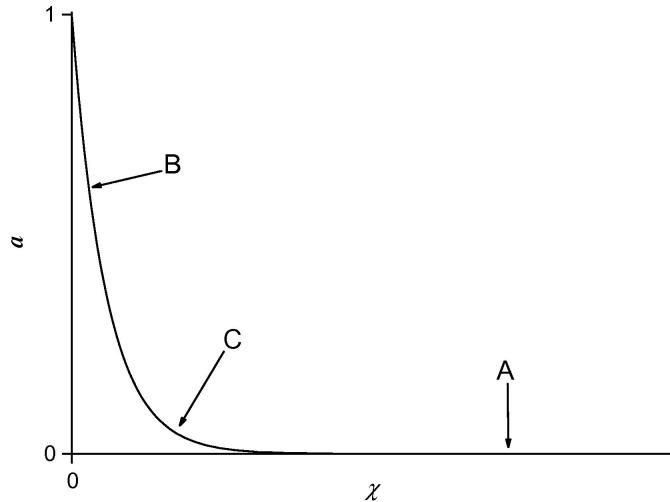
Concentration profile showing the regions where a high mesh density is required. In region A, the concentration profile *a* is horizontal, here only a small number of points, *i.e.* a low mesh density, is sufficient. In region B, the gradient is high, so a higher mesh density is required. In region C, the gradient of the concentration profile is changing rapidly. This also requires a high mesh density.

When performing electrochemical simulations using finite difference methods a useful reduction in the number of points required for a given accuracy (and hence increase in the speed of the simulation) can be achieved by using a non-linear mesh spacing [28]. This concentrates the computational effort in those regions where the concentrations and fluxes change most rapidly. This is normally achieved by using a high mesh density, *i.e.* closely spaced points, where the concentration gradient is steepest. For simple electrochemical systems such as the ec or the ec′ mechanisms, or where a rotating disc electrode is used, the concentration profile is steepest at the electrode surface. Thus Feldberg [29] used a mesh spacing based on an error function to give a close point spacing at the electrode, with the spacing increasing with distance from the electrode in an appropriate way. This approach, and similar methods, are widely used. The problem with this type of pre-defined mesh spacing is that it requires an *a priori* knowledge of the approximate shape of the concentration profiles. In the present case [1], the concentration profiles may be steep at the electrode surface, at the membrane–solution interface, at both, or even at some point in between depending on the balance between the different rate processes. It has also been found that a high mesh density is required where the concentration gradients are rapidly changing, *i.e.* where d^2^[A]/d*x* ^2^ or d^2^[S]/d*x* ^2^ are large. For concentration profiles such as those shown if Fig. 2 , this requires a higher mesh density at some region within the film that is not necessary near the boundaries. The relaxation method allows the optimum mesh spacing to be achieved automatically [16] by deriving an equation describing the relationship between the mesh density and the concentration profiles. This equation is then solved simultaneously along with Eqs. (4) and (5).

**Fig. 3:**
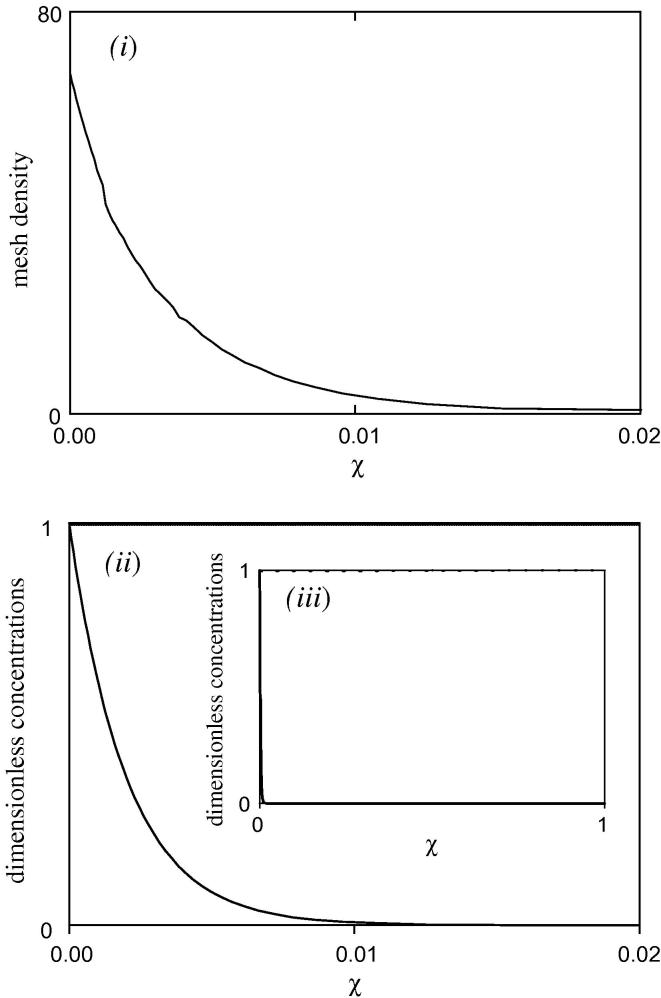
Graphs showing the advantages of automated mesh point allocation. The full concentration profile is shown in graph (iii). The solid line is the concentration profile for A (=**y**_1_), the dashed line that for S (=**y**_2_). Graph (ii) shows the profile near the electrode surface. The effect of the automated mesh point allocation is apparent. Of the 200 points covering the distance 0 ⩽ *χ* ⩽ 1, 42 points are in the region 0 ⩽ *χ* ⩽ 0.01, *i.e.* 21% of the points in 1% of the distance. This is also shown by graph (i) showing the mesh density, d**q**/d*χ* (=*h*/Δ**y**_7_) which varies from 68 at *χ* = 0 to 0.78 for 0.04 ⩽ *χ* ⩽ 1. The average mesh density is 1. If a linear point spacing were used, of the 200 points only 2 would lie in the region 0 ⩽ *χ* ⩽ 0.01. Simulated for *κ* = 500, *γ* = 10^−5^, *η* = 1, *μ* = 0.001, *a*_*ε*_ = 1, with weighting parameters, *P*_1_ = 50, *P*_2_ = 2.5, *P*_3_ = 2.5, *P*_4_ = 100, *P*_5_ = 100.

**Fig. 4:**
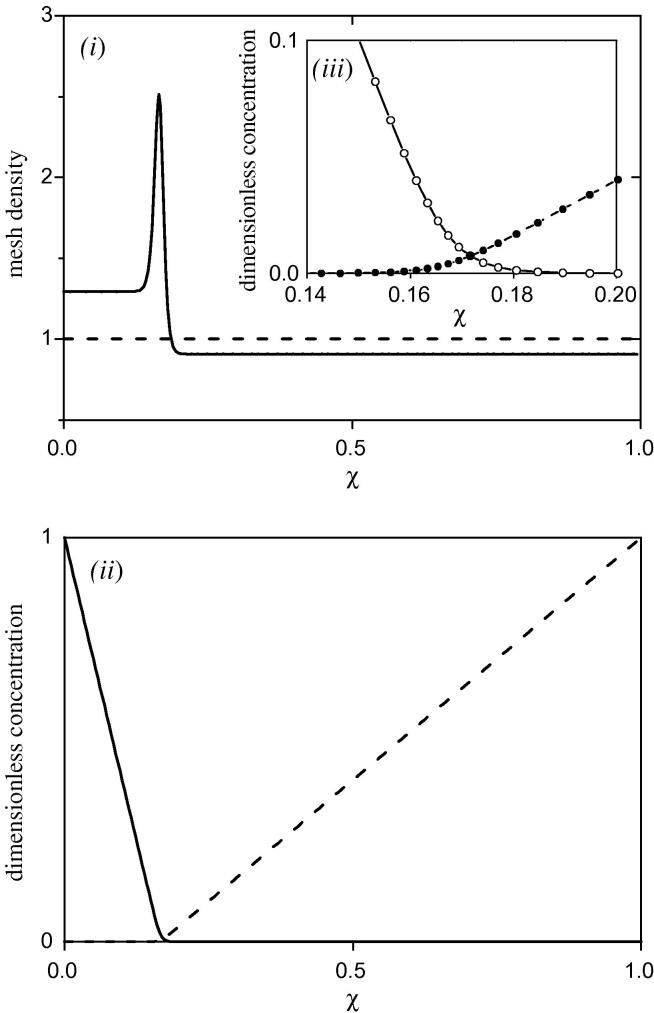
Graphs showing the advantages of considering the second order differentials of the concentration profiles in the mesh density function. The full concentration profile is shown in graph (ii). The solid line is the concentration profile of A (=**y**_1_), the dashed line that of S (=**y**_2_). Graph (i) shows the mesh density, d**q**/d*χ* (=*h*/Δ**y**_7_). The mesh density is higher near to the inner boundary than it is near to the outer boundary, due to the relative gradients of *a* and *s*. The peak in the mesh density at *χ* = 0.017 is due to *P*_4_ and *P*_5_ in Eq. (25). This gives a high mesh density where the gradients of the concentration profiles change rapidly, i.e. at the titration point. The effect of this is shown by graph (iii), which shows the region of the concentration profile around the titration point. The distance 0.16 ⩽ *χ* ⩽ 0.18 contains 8 points, compared with an average of approximately 4 points over the same distance in the rest of the film. Simulated for *κ* = 200, *γ* = 0.2, *η* = 1, *μ* = 0.001, *a*_*ε*_ = 1, with weighting parameters, *P*_1_ = 50, *P*_2_ = 2.5, *P*_3_ = 2.5, *P*_4_ = 100, *P*_5_ = 100.

Figs. 3 and 4 demonstrate the benefits of automated mesh point allocation. In Fig. 3, as described in the legend, a linear mesh spacing would not accurately describe the profile, since the reaction layer only covers approximately 1% of the film, near the electrode surface. A pre-defined mesh spacing with, for example, mesh density decreasing exponentially with *χ*, would be better than nothing, but, since the substrate profile may be steep near *χ* = 1, a high mesh density would also be required there. This would be wasteful of points, and computationally inefficient, if the concentration profiles are only steep at one of the boundaries.

Fig. 4 shows the effect of making the mesh density depend on the second differential of the concentration profiles, *i.e.* the rate of change of the gradient. Here a high mesh density is required at a position away from the boundaries. Since this may occur anywhere within the film, a single pre-defined mesh spacing function would not be useful.

Both the relaxation and Simplex methods and their implementation are described in detail by Press et al. [16]. In this paper we only describe how these methods are used to simulate the immobilized enzyme electrode and to analyse experimental data. This should be sufficient to allow the reader to adapt the program for other model systems. Our programs make use of the subroutines published by Press et al. [16] and are available from the authors on request.

### 3.1 Implementation of the relaxation method

#### 3.1.1 Representation of concentration profiles

In our system, four variables are required to describe the concentration profiles of mediator and substrate

(18) $$y_{1}=a$$

(19) $$y_{2}=s$$

(20) $$y_{3}=\frac{da}{d\chi }$$

(21) $$y_{4}=\frac{ds}{d\chi }$$

Then $\frac{d^{2}a}{d\chi^{2}}$ is represented as the first order differential equation $\frac{dy_{3}}{d\chi }$.

#### 3.1.2 Automated mesh point allocation

The relaxation method uses a grid of *N* mesh points to represent the distance 0 ⩽ *χ* ⩽ 1. Each variable **y**_**r**_ above is therefore an array of values where 0 ⩽ *n* ⩽ *N*.

In order to implement an adaptive non-linear mesh spacing we require three more **y**_*r*,*n*_ arrays:

(22) $$y_{5}=Q$$

(23) $$y_{6}=\frac{dQ}{dq}$$

(24) $$y_{7}=\chi$$

where **q** is equal to the point number *n*, *i.e.* it ranges from 1 to *N*. The variable **Q** is proportional to **q**, but does not have a defined range of values. The relationship between **Q** and distance *χ* at any one point is determined by the mesh density function $\Phi =\frac{dQ}{d\chi }$. A large value of **Φ** indicates that a high mesh density is required, *i.e.* a large number of points within a given distance Δ*χ*. The definition of **Φ** given by Press et al. [16] is not suitable for concentration profiles, so a new definition of **Φ** was used. A high mesh density is required where the concentration profiles are steep, **Φ** should therefore be proportional to d*a*/d*χ* and d*s*/d*χ*, so that it is large in, for example, region B of Fig. 2.

For our system, it was also found to be necessary to have a high mesh density where the gradient of the profile was rapidly changing, *i.e.* in region C of Fig. 2. If we analyse what Bartlett and Pratt [1] call the titration case, *i.e.* a situation where both mediator and substrate are consumed within the film resulting in a change from mediator to substrate limited kinetics through the film, there is a very sharp change in gradient at the titration point, *χ* ^∗^.

Taking account of these considerations, we find that a suitable definition of **Φ** is

(25) $$\Phi =P_{1}+P_{2}\left|\frac{da}{d\chi }\right|+P_{3}\left|\frac{ds}{d\chi }\right|+P_{4}\left|\frac{d(da/d\chi )}{dq}\right|+P_{5}\left|\frac{d(ds/d\chi )}{dq}\right|$$

which, in terms of the program variables **y**, becomes:

(26) $$\Phi =P_{1}+P_{2}|y_{3}|+P_{3}|y_{4}|+P_{4}\left|\frac{dy_{3}}{dq}\right|+P_{5}\left|\frac{dy_{4}}{dq}\right|$$

The variables *P*_1_ to *P*_5_ are weighting parameters, the larger the value the more effect that part of the equation has on the mesh spacing. The *P* variables do not need to be normalised, since the value of **Q** is automatically adjusted so that as 1 ⩽ *q* ⩽ *N* then 0 ⩽ *χ* ⩽ 1. Since it is the magnitude, and not the sign, of the differentials in Eq. (25) which determines the mesh spacing, absolute values are used.

With reference to Fig. 2, a high (relative) value of *P*_1_ favours a linear point spacing. A high value for *P*_2_ favours a high mesh density in region B. A high value of *P*_4_ favours a high mesh density in region C. *P*_3_ and *P*_5_ are the equivalents to *P*_2_ and *P*_4_ but for the substrate concentration profile.

The reason for using the method shown in Eqs. (25) and (26) instead of d^2^ *a*/d*χ* ^2^ and d^2^ *s*/d*χ* ^2^ for the second differentials, is that the latter would give the following expression

(27) $$\Phi =P_{1}+P_{2}|y_{3}|+P_{3}|y_{4}|+P_{4}\left|\frac{dy_{3}}{dy_{7}}\right|+P_{5}\left|\frac{dy_{4}}{dy_{7}}\right|$$

in which the expression for the mesh density, 1/Δ**y**_7_ (=1/Δ*χ*) occurs in the definition of the mesh density function, **Φ**. Errors in **y**_7_ would thus cause divergent behaviour.

The form of **Φ** shown in Eqs. (25) and (26) should be generally suitable for any system of concentration profiles.

#### 3.1.3 Differential equations

As described by Press et al. [16], *G* variables require *G* differential equations **W** to solve them. Two of these are given by the relationships between **y**_1_ and **y**_3_ and **y**_2_ and **y**_4_

(28) $$\frac{da}{dq}=\frac{da}{d\chi }\frac{d\chi }{dq}∴\;\frac{dy_{1}}{dq}=y_{3}\frac{dy_{7}}{dq}$$

similarly;

(29) $$\frac{dy_{2}}{dq}=y_{4}\frac{dy_{7}}{dq}$$

Two more are given by Eqs. (4) and (5):

(30) $$\frac{dy_{3}}{dq}=\frac{dy_{7}}{dq}\frac{\kappa^{2}y_{1}y_{2}}{\gamma y_{1}(1+\mu y_{2})+y_{2}}$$

(31) $$\frac{dy_{4}}{dq}=\frac{dy_{7}}{dq}\frac{\kappa^{2}y_{1}y_{2}}{\gamma y_{1}(1+\mu y_{2})+y_{2}}\frac{\gamma }{\eta }$$

From Eq. (22)

(32) $$\frac{dy_{5}}{dq}=y_{6}$$

As described earlier, there is a linear relationship between **q** and **Q**. Therefore

(33) $$\frac{dy_{6}}{dq}=0$$

The final differential equation is given by the mesh spacing function Φ

(34) $$\frac{dy_{7}}{dq}=\frac{y_{6}}{\Phi }$$

#### 3.1.4 Finite difference representation

These seven equations are expressed in finite difference form as **W**_*g*,*n*_, which couples two points *n* and *n* − 1, such that

(35) $$\frac{dy_{r\text{,}n-1/2}}{dq}\approx y_{r\text{,}n}-y_{r\text{,}n-1}=y_{r}^{m}$$

and

(36) $$y_{r\text{,}n-1/2}\approx \frac{y_{r\text{,}n}+y_{r\text{,}n-1}}{2}=1/2y_{r}^{p}$$

The abbreviations $y_{r}^{m}$ and $y_{r}^{p}$ are used to simplify the notation.

Using the notation shown to the right of Eqs. (35) and (36), the seven finite difference equations are therefore:

(37) $$W_{1\text{,}n}=y_{1}^{m}-(y_{7}^{m}y_{3}^{p})/2$$

(38) $$W_{2\text{,}n}=y_{2}^{m}-(y_{7}^{m}y_{4}^{p})/2$$

(39) $$W_{3\text{,}n}=y_{3}^{m}-\frac{\kappa^{2}y_{7}^{m}y_{1}^{p}y_{2}^{p}}{2(\gamma y_{1}^{p}(1+\mu y_{2}^{p})+y_{2}^{p})}$$

(40) $$W_{4\text{,}n}=y_{4}^{m}-\frac{\gamma \kappa^{2}y_{7}^{m}y_{1}^{p}y_{2}^{p}}{2\eta (\gamma y_{1}^{p}(1+\mu y_{2}^{p})+y_{2}^{p})}$$

(41) $$W_{5\text{,}n}=y_{5}^{m}-y_{6}^{p}h/2$$

(42) $$W_{6\text{,}n}=y_{6}^{m}$$

(43) $$W_{7\text{,}n}=y_{7}^{m}-\frac{y_{6}^{p}h}{2(P_{1}+1/2P_{2}|y_{3}^{p}|+1/2P_{3}|y_{4}^{p}|+P_{4}|y_{3}^{m}|+P_{5}|y_{4}^{m}|)}$$

where each **W**_*g*,*n*_ should be equal to zero for the correct solution. The variable *h* is equal to 1/(*N* − 1), *i.e.* it is the average point spacing Δ*χ*.

#### 3.1.5 Solution of the equations

For the correct solution, all **W**_*g*,*n*_ should be zero.

Taking all **W**_*g*,*n*_, **y**_*r*,*n*_, and **y**_*r*,*n*−1_ into consideration, Eqs. (15) and (16) become [16]:

(44) $$W_{g\text{,}n}(y_{r\text{,}n}+\Delta y_{r\text{,}n}\text{,}y_{r\text{,}n-1}+\Delta y_{r\text{,}n-1})\approx W_{g\text{,}n}(y_{r\text{,}n}\text{,}y_{r\text{,}n-1})+\overset{G}{\underset{r=1}{\sum }}(V_{g\text{,}r}\Delta y_{r\text{,}n-1}+V_{g\text{,}r+G}\Delta y_{r\text{,}n})$$

where

(45) $$V_{g\text{,}r}=\frac{\partial W_{g\text{,}n}}{\partial y_{r\text{,}n-1}}$$

(46) $$V_{g\text{,}r+G}=\frac{\partial W_{g\text{,}n}}{\partial y_{r\text{,}n}}$$

in which 1 ⩽ *r* ⩽ *G*, 1 ⩽ *g* ⩽ *G*, 1 ⩽ *n* ⩽ *N*.

Evaluation of all Δ**y**_*r*,*n*_ and Δ**y**_*r*,*n*−1_ therefore requires inversion of the **V** matrix. This is performed using subroutines published by Press et al. [16] and will not be discussed further here.

It can be seen from Eqs. (45) and (46) that the simulation requires prior evaluation of the differentials of every **V**_*g*,*n*_ with respect to every **y**_*r*,*n*_ and **y**_*r*,*n*−1 _. Thus with seven equations and seven variables, 98 differential equations are required. A few of these will be shown here as examples. For equation **W**_1_:

(47) $$V_{1\text{,}r}=\frac{dW_{1\text{,}n}}{dy_{r\text{,}n-1}}\text{;}\;1⩽r⩽7$$

For *r* = 1 we differentiate Eq. (37) with respect to variable **y**_1_ at point *n* − 1

(48) $$W_{1\text{,}n}=y_{1}^{m}-(y_{7}^{m}y_{3}^{p})/2$$

(49) $$V_{1\text{,}1}=\frac{d(y_{1\text{,}n}-y_{1\text{,}n-1}-(y_{7}^{m}y_{3}^{p})/2)}{dy_{1\text{,}n-1}}$$

(50) $$∴\;V_{1\text{,}1}=-1$$

For *r* = 2

(51) $$V_{1\text{,}2}=\frac{d(y_{1}^{m}-(y_{7}^{m}y_{3}^{p})/2)}{dy_{2\text{,}n-1}}$$

(52) $$∴\;V_{2\text{,}1}=0$$

For *r* = 3

(53) $$V_{1\text{,}3}=\frac{d(y_{1}^{m}-(y_{7}^{m}(y_{3\text{,}n}+y_{3\text{,}n-1}))/2)}{dy_{3\text{,}n-1}}$$

(54) $$∴\;V_{1\text{,}3}=-y_{7}^{m}/2$$

The remaining four **V**_1,*r*_ differentials are evaluated similarly. **V**_1,*r*+1_ to **V**_1,*r*+7_ are differentiated with respect to **y**_1_ at point *n*

(55) $$V_{g\text{,}r+G}=\frac{\partial W_{g\text{,}j}}{\partial y_{r\text{,}j}}\text{;}\;8⩽r+G⩽14$$

For example, **V**_1,14_ is **W**_1,*n*_ differentiated with respect to **y**_7,*n*_

(56) $$V_{1\text{,}14}=\frac{d(y_{1}^{m}-((y_{7\text{,}n}+y_{7\text{,}n-1})y_{3}^{p})/2)}{dy_{7\text{,}n}}$$

(57) $$∴\;V_{1\text{,}14}=-y_{3}^{p}/2$$

Thus we can see that many of the 98 differential equations are either 0, or yield a fairly simple expression so that the task is not so arduous as it might first appear.

A few of the equations are slightly more complicated, for example **V**_3,1_ is

(58) $$V_{3\text{,}1}=\frac{d\left(y_{3}^{m}-\frac{\kappa^{2}y_{7}^{m}y_{1}^{p}y_{2}^{p}}{2(\gamma y_{1}^{p}(1+\mu y_{2}^{p})+y_{2}^{p})}\right)}{dy_{1\text{,}n}}$$

Application of the quotient rule gives

(59) $$V_{3\text{,}1}=-\frac{\kappa^{2}y_{7}^{m}{\left(y_{2}^{p}\right)}^{2}}{2{\left(\gamma y_{1}^{p}(1+\mu y_{2}^{p})+y_{2}^{p}\right)}^{2}}$$

#### 3.1.6 Differentiation of absolute values

When determining **V**_7,3_, **V**_7,4_, **V**_7,10_ and **V**_7,11_ it is necessary to differentiate absolute values (recall the expression for **W**_7,*n*_ Eq. (43)). The strict mathematical way to do this is as follows

(60) $$\frac{d|f(x)|}{dx}=\mathrm{sign}(f(x))\frac{df(x)}{dx}$$

where sign(*f*(*x*)) is +1 for *f*(*x*) > 0, −1 for *f*(*x*) < 0 and 0 if *f*(*x*) = 0. However, in a limited number of cases this caused numerical problems when it was implemented. After extensive testing we found that by taking

(61) $$\frac{d|f(x)|}{dx}=\frac{df(x)}{dx}$$

we were able to obtain robust results for all cases (*note*: the use of Eq. (61) only affects the automated grid point allocation and it does not introduce any errors into the simulated concentration profiles or current, it only alters the efficiency of the simulation). Results obtained in this way are in good agreement with the analytical solutions corresponding to the different limiting cases. The equation used for **V**_7,3_ is therefore

(62) $$V_{7\text{,}3}=\frac{y_{6}^{p}h(1/2P_{2}-P_{4})}{2{\left(P_{1}+1/2P_{2}|y_{3}^{p}|+1/2P_{3}|y_{4}^{p}|+P_{4}|y_{3}^{m}|+P_{5}|y_{4}^{m}|\right)}^{2}}$$

This deals with the points between the electrode and the membrane–solution interface, 2 ⩽ *n* ⩽ *N* − 1. We next consider the boundaries.

#### 3.1.7 Boundary conditions

At the boundaries, the technique is similar. Since there is no *n* = 0 point, a differential equation for **y**_*r*,*n*−1_ is not required at the inner boundary where *n* = 1. Similarly, as there is no *N* + 1 point, the equation **W**_*g*,*n*_ is only differentiated with respect to y_*r*,*N*_. Therefore at the inner boundary *χ* = 0, *n* = 1:

(63) $$V_{g\text{,}r+G}=\frac{\partial W_{g\text{,}1}}{\partial y_{r\text{,}1}}$$

while at the outer boundary *χ* = 1, *n* = N:

(64) $$V_{g\text{,}r+G}=\frac{\partial W_{g\text{,}N+1}}{\partial y_{r\text{,}N}}$$

where 1 ⩽ *r* ⩽ *G*. Thus there are potentially a total of 2*G* boundary conditions.

To solve *G* equations, *G* boundary conditions must be known. There does not necessarily need to be one boundary condition specific to each **y** array, *i.e.* some **y** arrays may have both boundary conditions known, while others have none. For our problem, four boundary conditions are known for **y**_1_ to **y**_4_. At the inner boundary *χ* = 0, *n* = 1

(65) $$a=a_{\varepsilon }\text{;}\;W_{7\text{,}1}=y_{1\text{,}1}-a_{\varepsilon }\text{;}\;V_{7\text{,}8}=1$$

(66) $$\frac{ds}{d\chi }=0\text{;}\;W_{6\text{,}1}=y_{4\text{,}1}\text{;}\;V_{6\text{,}11}=1$$

At the outer boundary *χ* = 1, *n* = *N*:

(67) $$\frac{da}{d\chi }=0\text{;}\;W_{1\text{,}N+1}=y_{3\text{,}N}\text{;}\;V_{1\text{,}10}=1$$

The substrate concentration has its bulk value

(68) $$s=1\text{;}\;W_{2\text{,}N+1}=y_{2\text{,}N-1}\text{;}\;V_{2\text{,}9}=1$$

Three more boundary conditions are required, these relate to the mesh spacing. At the inner boundary

(69) $$Q=0\text{;}\;W_{5\text{,}1}=y_{5\text{,}1}\text{;}\;V_{5\text{,}12}=1$$

(70) $$\chi =0\text{;}\;W_{4\text{,}1}=y_{7\text{,}1}\text{;}\;V_{4\text{,}14}=1$$

At the outer boundary

(71) $$\chi =1\text{;}\;W_{3\text{,}N+1}=y_{7\text{,}N-1}\text{;}\;V_{3\text{,}14}=1$$

**Fig. 5:**
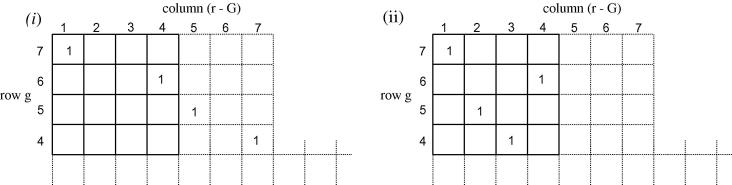
Diagram showing the structure and position of the pivot elements in the **S**_*g*,*r*_ matrix. The pivot elements are indicated by the solid lines. The elements containing a ‘1’ are due to the boundary conditions, Eqs. (65)–(71). All other elements are equal to zero. For pivoting to work, each of the first four columns (1 ⩽ *r* − *G* ⩽ 4) must contain at least one non-zero element within its first four rows (4 ⩽ *g* ⩽ 7) [16]. The matrix (i) shows the initial arrangement of the columns. It can be seen that columns 2 and 3 contain only zeros in their first four rows. Swapping column 2 with 5, and column 3 with 7 corrects this, as shown in matrix (ii).

All other **V**_*g*,*r*_ are zero. Note that the ordering of the index *g* in the equations **W**_*g*,*n*_ is arbitrary. The ordering of the **V** values within the matrix is however vitally important. Due to the method used to invert the matrix, the first 4 × 4 block is used for ‘pivoting’ and must not be singular for the reasons described by Press et al. [16]. What this means is that each of the first four columns (1 ⩽ *r* − *G* ⩽ 4) of the matrix **V**_*g*,*r*_ must contain at least one non-zero value within its four rows (4 ⩽ *g* ⩽ 7). This is shown diagrammatically in Fig. 5 and is explained in detail by Press et al. [16].

#### 3.1.8 Fluxes

Once the correct values of the **y** arrays have been found by the simulation, the values of the dimensionless mediator and substrate fluxes are given by

(72) $$J_{\text{A}}=-{\left(\frac{da}{d\chi }\right|}_{\chi =0}=-y_{3\text{,}1}$$

(73) $$J_{\text{S}}=-\frac{\eta }{\gamma }{\left(\frac{ds}{d\chi }\right|}_{\chi =1}=\frac{\eta }{\gamma }y_{4\text{,}N+1}$$

where *J*_A_ is the dimensionless form of the flux measured at the electrode.

### 3.2 Simplex algorithm

The Simplex algorithm [16,30] was used to determine the parameter values which gave the best fit of the theoretical function to the experimental data. The algorithm calculates the goodness of fit using the equation of minimum squares

(74) $$\zeta^{2}=\overset{\Omega }{\underset{i=1}{\sum }}{\left({\text{Data}}_{\exp}-{\text{Data}}_{\text{theo}}\right)}^{2}$$

where *Ω* is the number of experimental data points and then seeks to minimize *ζ* ^2^.

This fitting algorithm requires a set of starting values for the parameters to be fitted. We generate these by fitting of our experimental data to the approximate analytical expressions [1] that describe the system. In a subsequent paper, we will present a full experimental data set and give a detailed description of the concerted approach used to analyse all of the data combining the numerical simulation methods described here with fitting to the approximate analytical expressions.

### 3.3 Incorrect solutions

One potential problem with the relaxation method is that there may be more than one mathematical solution which satisfies the set of equations and boundary conditions. With a transient simulation method, since it proceeds stepwise from the initial conditions, it mimics the physical behaviour of the system. Such a method, correctly implemented, should therefore reach the correct physical solution to the system. Steady state simulations, however, do not follow the physical behaviour of the system, and will converge on the first mathematically correct, or approximately correct, solution which they encounter. Usually if mathematically correct solutions other than the required physical solution exist, these will not correspond to physically possible solution, for example they may give negative concentrations or normalized concentrations greater than 1. Such solutions can therefore be rejected, and the simulation repeated using different starting conditions in order to look for the correct solution. Routines were implemented in the simulation program to avoid such impossible solutions from occurring. A subroutine operates on *y*_1,*n*_ and *y*_2,*n*_ and sets them equal to zero if they become negative, or sets them to 1 if they exceed 1. A second subroutine ensures that the distance must always increase from the electrode surface to the membrane–solution interface. This prevents Δ*y*_7,*n*_ from becoming negative during the automated mesh point allocation, this is achieved by checking that *y*_7,*n*_ is greater than *y*_7,*n*−1_, if this is not true then *y*_7,*n*_ is set equal to *y*_7,*n*−1_. This results in a step in the **y**_7_ array, from which the simulation can recover to give a smooth **y**_7_.

For the Simplex method, there also may be more than one set of parameters that minimizes *ζ* ^2^, *i.e.* there can be various local minima. It is therefore important to apply sensible constraints on the values of the fitting parameters. These constrains usually derive from three different origins: (1) experimental knowledge about a particular system; (2) approximate values obtained using approximate analytical solutions to which the experimental results can also be fitted; (3) comparison of the fitting parameters obtained when different experimental variables are independently modified for a given system. Therefore, the model is not only simulating the experimental response but also predicting its evolution.

In practice, it is sometimes very difficult to find physically meaningful results when trying to fit three or more parameters at the same time. In these cases it is usually more convenient to start by fixing, or tightly constraining, one parameter until approximate fitting results have been found for the more uncertain ones.

Finally, it is essential to critically evaluate the progress and results of the fitting to check that the resulting parameters are consistent and physically sensible.

### 3.4 Accuracy and limitations of the simulations

The default values for the weighting parameters used in most of our simulations (particularly in all the simulations shown in this paper) are *P*_1_ = 50, *P*_2_ = *P*_3_ = 2.5 and *P*_4_ = *P*_5_ = 100. With the correction subroutines described above and automated mesh point allocation, the simulation works reliably up to *κ* ≈ 1000, for *γ*/*η* < 1, or *κ*/*η* ^1/2^ ≈ 1000 for *γ*/*η* > 1. This combination of parameters covers all of the physically reasonable situations that could occur for our electrodes. The higher values of *κ* correspond to films that are much thicker than any we are able to achieve experimentally. Over this range of *κ*, *η*, and *γ* the simulation was in excellent agreement with the approximate analytical solutions with the largest deviations found at the case boundaries where the approximate analytical solutions are least accurate.

Unlike transient simulation methods, the accuracy of the relaxation method does not suffer when high rates of reaction, i.e. large *κ* or *κ*/*η* ^1/2^ are used. The simulation simply reaches a point at which it can no longer converge.

Calculations were carried out on a Celeron^®^ CPU 2.80 GHz, 448 MB of RAM personal computer using a program written in FORTRAN 77 and incorporating the algorithms for the relaxation and Simplex methods given by Press et al. [16]. For our problem, with a set of seven ordinary differential equations and 200 grid points, each iteration takes less than 1 s and typically less than 100 iterations are required to achieve an acceptable solution.

## 4 Experimental

### 4.1 Reagents and materials

The following chemicals were used without further purification: sodium 3-mercapto-1-propanesulfonate (MPS; Aldrich), glucose (Merck) and TRIZMA base and Tris–HCl (Sigma). Doubly distilled water was purified with a Milli-Q^®^ reagent water system (Millipore). Aqueous solutions of glucose oxidase (EC 1.1.3.4), from *Aspergillus niger* were prepared from Fluka reagent without further purification. The complex [Os(bpy)_2_Cl(PyCOH)]Cl (where PyCOH is pyridinecarbaldehyde) was prepared as previously reported, osmium poly(allylamine) (PAH-Os) was synthesized as described elsewhere [31].

Gold coated silicon (1 0 0) substrates were employed as electrodes with a 20 nm titanium and a 20 nm palladium adhesion layer and a 200 nm gold layer thermally evaporated using an Edwards Auto 306 vacuum coating system, at *p* < 1 × 10^−5^ mbar. The freshly evaporated gold film substrates were used once only. To check the quality of the gold surface, the electrodes were cycled in 2 M sulphuric acid between 0 and 1.6 V at 0.1 V s^−1^.

*Surface modification*. An automatic dipping method (Microm DS50 programmable slide stainner from Zeiss Inc.) was used to implement the process described by Hodak et al. [23] to build up layer-by-layer supramolecular multilayers comprised of GOx and PAH-Os. First, the gold surface was modified with sulphonate groups by immersion in a freshly prepared 20 mM 3-mercapto-1-propane sulphonic acid (MPS) solution for 30 min followed by rinsing with Milli-Q^®^ water. After thiol adsorption, the first PAH-Os layer was formed by immersion of the thiol-modified Au substrate in a PAH-Os solution for 10 min. The next and subsequent layers were deposited onto the modified surface by alternated immersions in a 1 μM GOx solution and PAH-Os solution respectively for 10 min each, thoroughly rinsing in Milli-Q^®^ water at the end of each adsorption step.

A standard three-electrode electrochemical cell was employed with an operational amplifier potentiostat (TEQ-Argentina). A Ag/AgCl; 3 M KCl reference electrode was used together with a large area platinum gauze counter electrode. All electrochemical experiments were carried out in deoxygenated 0.1 M TRIS buffer solutions (0.2 M ionic strength) of pH 7.4, at room temperature (25 ± 2) °C. A SENTECH (Berlin, Germany) variable angle rotating-analyzer automatic ellipsometer (vertical type, 2000 FT model) equipped with a 632 nm laser as polarized light source was employed to measure the film thickness of the electrodes.

## 5 Comparison of the simulations to experimental data

We chose to test our numerical simulations on electrostatically self assembled enzyme electrodes. The GOx/PAH-Os system [22] is a previously well characterized one. It has been extensively studied by cyclic voltammetry, quartz-crystal microbalance, ellipsometry, FT-IR and Raman spectroscopy [32–36]. We know that film thickness, Os surface concentration and enzyme loading all increase with the number of adsorption steps and the catalytic current varies with the film thickness. It has been established that the redox charge propagation within the film is by electron hopping and the diffusion coefficient has been estimated [37]. We know that we can approximate the substrate partition coefficient between the solution and the film, *K*_s_, to unity. Finally, we know that, because of the high water content of the films, the glucose diffusion coefficient within the film is almost the same as in pure water [35].

The great advantage of electrostatically self-assembled systems as compared to other enzyme electrodes, such as hydrogels, is that we can vary the design of our electrodes at will choosing from a more or less wide spectrum the thickness, enzyme loading and Os concentration; and, to a lesser extent, *k* the enzyme–mediator reoxidation rate constant. In this way, we are able to test the simulations on electrodes covering a wide range of parameters, and not only on one or two specific cases.

There are five adjustable parameters, *κ*, *γ*, *η*, *μ*, and *a*_*ε*_, in our model (the values *P*_1_–*P*_5_ which control the automated grid point allocation are held fixed). Of these five, typically only two or three will be well determined by the curve fitting for any given set of experimental data from a single experiment. Which two or three parameters this is will depend on the case in which the experiment falls. To determine all five parameters with acceptable accuracy the results from more than one experiment carried out over a range of conditions such as film thickness, enzyme loading, mediator concentration corresponding to different cases would need to be combined. We will return to this point in a subsequent paper.

**Fig. 6:**
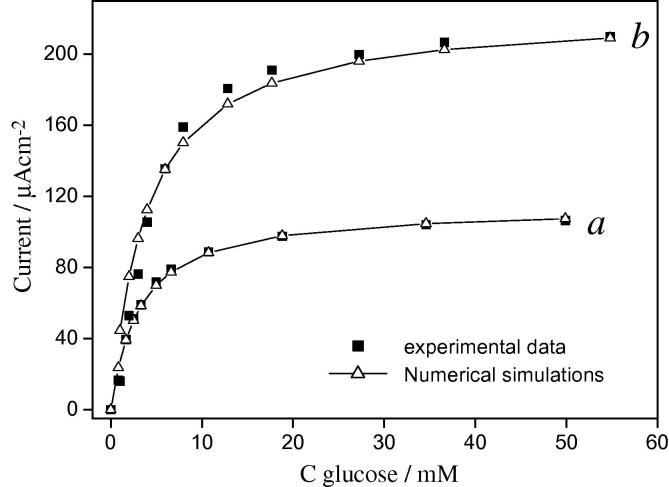
Graph showing experimental data and numerical simulations for amperometric response in the presence of β-d-glucose for two different electrodes. Both electrodes were prepared by electrostatic self-assembly of GOx and PAH-Os on a MPS-modified gold electrode. The numerical simulations used the relaxation method to solve the differential equations for the concentrations. A fitting routine using the Simplex algorithm was used to find the best fitting parameters for [*E*_wired_] and *k*: (a) MPS/(PAH-Os/GOx)_3_ electrode; (b) MPS/(PAH-Os/GOx)_4_ electrode.

Fig. 6 compares the results of the numerical simulations for two different sets of experimental data for the amperometric response as a function of substrate concentration for two self-assembled electrodes of different thickness. Table 1 summarises the numerical results.

Curve *a* is data for a MPS/(PAH-Os/GOx)_3_ electrode. The film thickness measured by ellipsometry was 141 nm. By analyzing the concentration profiles (see Supporting Information) and the ratio of the kinetic constants to the diffusion coefficients, we know that the data points can be fitted to the approximate analytical solution for the thin layer model [1,23]

(75) $$i=\frac{2{\mathrm{Fk}}_{\text{cat}}\Gamma_{\text{we}}}{1+\frac{k_{\text{cat}}}{k{\left[\text{A}\right]}_{\text{TOT}}}+\frac{K_{\text{MS}}}{{\left[\text{S}\right]}_{\infty }}}$$

where *i* is the current density, *F* is the Faraday, *Γ*_we_ is the wired enzyme surface concentration and the other parameters have been defined above. Fitting to the thin layer model has been used before to extract *k* and *Γ*_we_ from calibration plots.

This set of data is particularly useful to test the numerical simulations since we can compare the parameters resulting from the combined relaxation/Simplex fitting to the parameters obtained from fitting to the approximate analytical formula that has been previously validated experimentally [23].

When appropriately constrained, the relaxation/Simplex fitting gives the same fitting parameters as a non-linear fitting to Eq. (75). We can see from Fig. 6, that the simulations match our experimental data.

Having tested the approach on a data set for which there is a good analytical approximation we now go on to use it to analyse a data set for which the approximate analytical approach does not work. Curve ‘b’ in Fig. 6 depicts another set of experimental data for a MPS/(PAH-Os/GOx)_4_ electrode. In this case, the film thickness measured by ellipsometry was 222 nm. When performing the same analysis as for the data in curve ‘a’ (see Supporting Information), we realize that we cannot fit this data to the thin layer model or any other approximate analytical equation. This is one particular case where the numerical simulations could be of great help in trying to find the unknown parameters for our experimental system. Again from Fig. 6 we can see that there is good agreement between our experimental data and our simulations.

If we compare the parameters obtained for the two electrodes (shown in Table 1), we see that [*E*_wired_] for the two films differs by about 20%, but that the two *k* values are quite different. This can be explained if we consider that in electrostatically assembled multilayer films, the first layers are more strongly affected by the substrate so that thinner films may be expected to show different behaviour from their thicker analogs.

## 6 Conclusions

We have proposed, and described in detail, a new numerical simulation method to simulating the steady-state coupled reaction–diffusion problem for immobilized enzyme electrodes. We have investigated the advantages of the relaxation method with non-linear automated mesh spacing over more commonly applied numerical techniques for problems of this type.

The relaxation method is a very powerful tool that could be applied to a wide variety of electrochemical systems, provided that some form of bounded system can be set up, either in the form of a membrane or by enhanced mass transport such as convection or use of a microelectrode.

We have combined the relaxation simulation technique with the Simplex fitting algorithm and used the fitting routine to simulate the amperometric response of self-assembled GOx/PAH-Os electrodes to extract the unknown kinetic parameters from experimental data for the steady-state current at different glucose concentrations. The simulated currents are shown to be in excellent agreement with the experimental results. One of the data sets allowed us to compare the fitted parameters from our relaxation/Simplex fitting routine with parameters obtained by fitting the same data to an approximate analytical formula for the thin layer model. The two methods where shown to be in good agreement.

For the second set of experimental data, there was no unique analytical formula valid for all our data points. In this case, the fitting routine proved useful for finding unknown parameters that characterize our system.

## Figures and Tables

**Table:** 

Symbol	Definition
A (B)	oxidised (reduced) mediator
S (P)	substrate (product) of the enzymatic reaction
E	enzyme
E_1_ (E_2_)	enzyme in oxidized (reduced) state
[A]	oxidised mediator concentration
[S]	substrate concentration
[E]_TOT_	total enzyme concentration
*K*_MS_	Michaelis constant
*k*_cat_	catalytic rate constant
*k*	mediation rate constant
[A]_TOT_	total mediator concentration
[S]_∞_	substrate concentration in bulk solution
*D*_*i*_	diffusion coefficient for species *i*
*L*	thickness of the film
*K*_*i*_	partition coefficient between film and solution for species *i*
*x*	distance to the electrode
*a*	dimensionless oxidised mediator concentration
*s*	dimensionless substrate concentration
*κ*, *η*, *γ*, *μ*	dimensionless parameters describing a particular system
*χ*	dimensionless distance to the electrode surface
**y**	variable
**W**	differential equation
*n*	point number; (1 ⩽ *n* ⩽ *N*)
*N*	number of points
*g*	variable number (1 ⩽ *g* ⩽ *G*)
*r*	equation number; (1 ⩽ *r* ⩽ *G*)
**V**	matrix of equations
*h*	average point spacing
**q**	mesh point coordinate
**Q**	variable mesh coordinate
**Φ**	mesh spacing function
*P*	weighting parameter
*Ω*	number of data points
*J*_*i*_	flux of species *i*
*ζ*	function minimized by Simplex algorithm
*i*	current density
*Γ*_we_	surface concentration of wired enzyme
*F*	Faraday

**Figure S-1:**
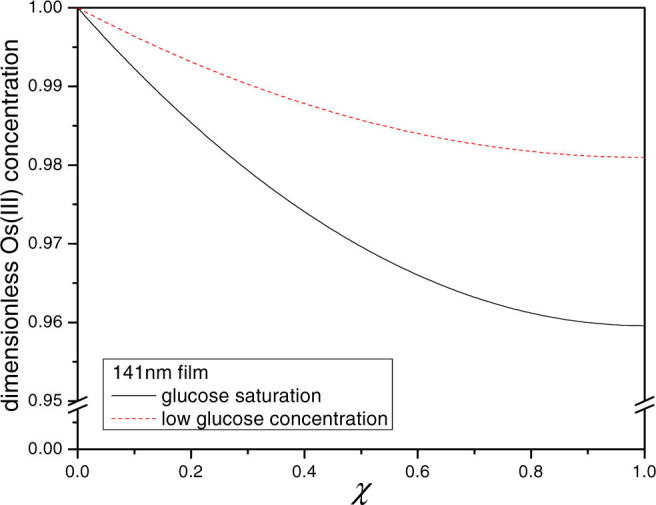
Oxidized mediator concentration profiles, decaying from the Nernst concentration at the electrode surface. The decay is minor both at low (red curve) and high (black) glucose concentration and hence the thin film approximation is still valid. Results for the 141nm electrode (curve *a* in Fig. 6). Black curve simulated for *κ* = 0.3194, *γ* = 0.0757, *η* = 262.7, *μ* = 2, *a*_*ε*_ = 1. Red curve simulated for *κ* = 0.3194, *γ* = 1.5147, *η* = 262.7, *μ* = 0.1, *a*_*ε*_ = 1. Weighting parameters for both curves, *P*_1_ = 50, *P*_2_ = 2.5, *P*_3_ = 2.5, *P*_4_ = 100, *P*_5_ = 100.

**Figure S-2:**
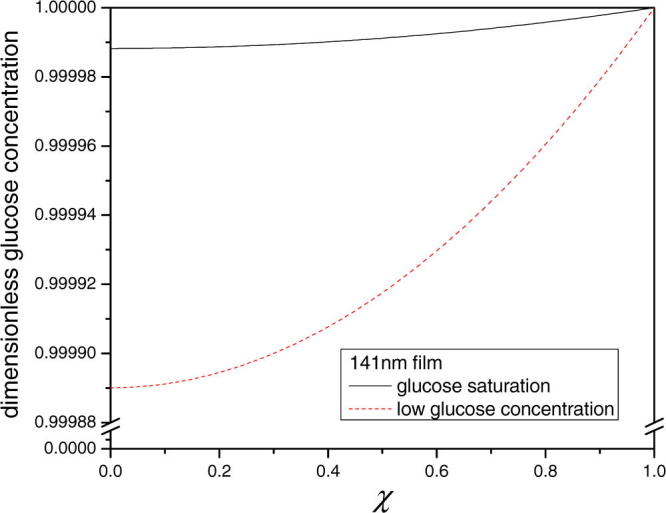
Glucose concentration profiles, decaying from the bulk solution concentration at the outside of the film. The decay is minor both at low (red curve) and high (black curve) glucose concentration and hence the thin film approximation is still valid. Dimensionless and weighting parameters are the same as in figure S-1.

**Figure S-3:**
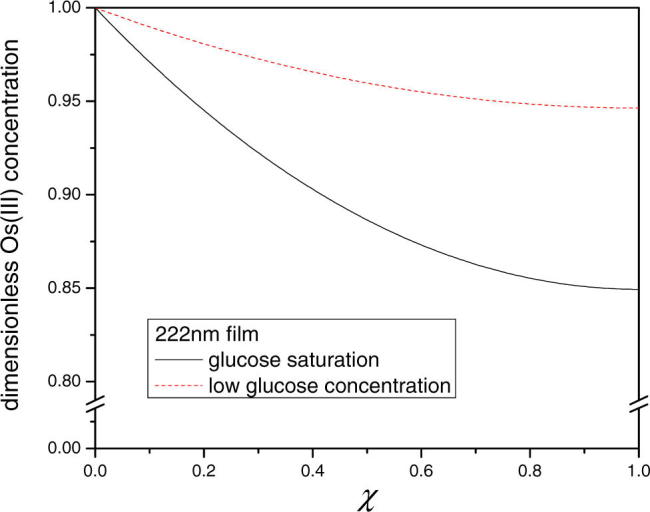
Oxidized mediator concentration profiles, decaying from the Nernst concentration at the electrode surface. The decay is much more noticeable than in the previous and thinner film. It is of particular importance at high (black curve) glucose concentration. Red curve is low glucose concentration. Hence the thin film approximation starts to break down at high glucose concentration. This effect would be even more remarkable in thicker films (more adsorption steps). Results for the 222 nm electrode (curve b in Figure 6). Black curve simulated for *κ* = 0.6991, *γ* = 0.0798, *η* = 644.5, *μ* = 4, *a*_*ε*_ = 1. Red curve simulated for *κ* = 0.6991, *γ* = 3.1900, *η* = 644.5, *μ* = 0.1, *a*_*ε*_ = 1. Weighting parameters for both curves, *P*_1_ = 50, *P*_2_ = 2.5, *P*_3_ = 2.5, *P*_4_ = 100, *P*_5_ = 100.

**Figure S-4:**
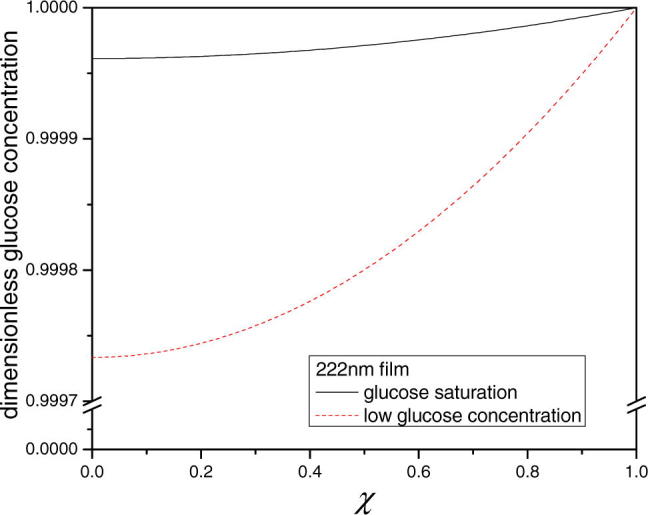
Glucose concentration profiles, decaying from the bulk solution concentration at the outside of the film. Though the decay is more important both at low (red curve) and high (black curve) glucose concentration than in the thinner electrode, these are not the results that make the thin film approximation break down, as is the case for the concentration of Os(III). Dimensionless and weighting parameters are the same as in figure S-3.

**Table 1:** Parameters obtained from the simulations in Fig. 6

Curve in Fig. 6	Thickness/nm	*k*/M^−1^ s^−1^	[*E*_wired_]/mM
a	141	562	1.096
b	222	1379	0.863

## Appendix 1

Appendix 1 List of symbols used throughout the text
